# Outcomes of screening and surveillance in people with two parents affected by colorectal cancers: experiences from the Familial Bowel Cancer Service

**DOI:** 10.1186/s13053-019-0122-8

**Published:** 2019-08-16

**Authors:** Jennifer Pan, Masha Slattery, Natalie Shea, Finlay Macrae

**Affiliations:** 1 Department of Colorectal Medicine and Genetics, Royal Melbourne Hospital, Level 1 South, 300 Grattan Street, Parkville, Victoria 3050 Australia; 2 Department of Medicine, Division of Gastroenterology and Hepatology, Stanford University, School of Medicine, 3801 Miranda Ave., Suite GI-111, Palo Alto, CA 94304 USA

**Keywords:** Colon cancer, Genetic risk factors, Cancer screening

## Abstract

**Background:**

The Familial Bowel Cancer Service at The Royal Melbourne Hospital was started in 1980 in order to offer bowel cancer screening services to those felt to be at a higher risk of CRC due to their family history, and upon registration in this service, patients gave consent for recording of their individual and familial medical history as pertaining to colorectal cancer in the FamBIS database. Using the FamBIS database, we sought to understand whether the subpopulation of individuals in whom both parents were diagnosed with colorectal cancer carried a higher risk of colorectal cancer or neoplastic polyps and should therefore undergo more intensive screening above that of the average-risk individual.

**Methods:**

We conducted a single-centre retrospective cohort-study of adults (18 years of age and older) in the FamBIS database, with review of their medical histories as pertaining to CRC diagnosis, screening, and surveillance from 1980 to 2015.

**Results:**

We identified and reviewed the medical histories of 96 registrants from 62 unique families. Registrants began screening as early as 24 years of age, with the mean age of first screening being at 44.6 ± 10.7 years old. The mean duration of screening was 17.3 ± 10.1 years, and through their screening period, registrants underwent an average of 11.5 ± 9.1 FOBTs and 4.4 ± 3.1 colonoscopies or sigmoidoscopies.

Over the course of screening, 41 (42.7%) registrants were found to have at least one neoplasm of any kind (including adenomas, advanced adenomas, and CRC) as their first positive colonoscopic finding. In total, 12 (12.5%) of the registrants were found to have an advanced neoplasm over the course of screening and surveillance, while only 2 patients were found to be diagnosed with CRC.

**Conclusions:**

The prevalence rates for neoplasms, advanced neoplasms, and CRC in our current study were statistically significantly higher compared with those seen in average-risk populations. This supports the importance of more intensive screening for this subpopulation in preventing colorectal cancers, as well as pre-and early-cancerous neoplasms.

## Background

Bowel or colorectal cancer [CRC] is the second-most diagnosed malignancy as well as second-most common cause of cancer-related death in Australia, with approximately 16,000 incident cases annually and estimated to account for 11% of all deaths from cancer in 2019 [1, 2]. Known risk factors for CRC include age, personal history of inflammatory bowel disease, personal or family history of colorectal cancer or polyps, as well as lifestyle factors like obesity, diet, and tobacco or alcohol use. Screening and surveillance have been partially credited for the decreasing trends in incidence and mortality associated with CRC by early diagnosis of cancers or removing pre-cancerous polyps [3]. Several modalities for CRC screening have been developed over the years, the main goal of which is cancer prevention (through detection and removal of pre-malignant polyps) and early detection of colorectal cancers. The major modalities currently in use worldwide include endoscopic techniques such as colonoscopy and sigmoidoscopy, radiologic testing including CT colonography and double-contrast barium enema, and stool testing including fecal occult blood/fecal immunochemical testing [FOB/FIT], with the newest being stool DNA testing.

Family history of colorectal cancer is often used as a determinant of risk of advanced adenoma or adenoma multiplicity [4–7]. Traditionally, guidelines for CRC screening in Australia and other countries have been based upon a person’s family history to determine his/her personal risk of developing CRC and therefore need for and recommended modality for screening [8, 9]. In mid-2006, the Australian Department of Health launched the National Bowel Cancer Screening Program—a population-based screening program using a stool-based immunochemical Faecal Occult Blood Test [iFOBT]. In the first step of the CRC screening protocol, Australians are encouraged to speak with their general practitioner [GP] to review their personal colorectal cancer risk, with risk assessment involving discussion of symptoms and family history.

Risk quantification based on family history then places individuals in one of three categories of relative risk: Category 1 – those at or slightly above average-risk, Category 2 – those at moderately increased risk, and Category 3 – those at potentially high risk. The previous 2005 National Health and Medical Research Council [NHMRC]-approved clinical guidelines used quantification criteria which focused primarily on the identification of affected family members with young-onset (defined as before the age of 55) or those who have multiple affected family members on one side of the family tree [10]. Those in Category 1 were recommended for average-risk screening, such as with iFOBT, as well as consideration of sigmoidoscopy, with colonoscopy only advised in symptomatic patients. Those deemed to have significant family history based on family history criteria (Category 2 and 3) were recommended to undergo more intensive screening protocols including colonoscopy or sigmoidoscopy with CT colonography or double-contrast barium enema. With these guidelines, however, the subset of the population in which both parents of an individual have been diagnosed with CRC were classified into Category 1 (unless one or more parents was diagnosed before the age of 55). As such, recommendations for screening in this population did not previously include colonoscopy, but the most recent updated 2017 guidelines, however, do include recommendations for colonoscopy in this population [11].

Our study evaluated a special subset of the population in which both parents of an individual have been diagnosed with CRC. Under previous Australian guidelines, individuals in this population would not qualify as moderately-increased risk with recommendation of colonoscopic screening. We sought to understand whether this population carried a higher risk of colorectal cancer or neoplastic polyps and should therefore undergo more intensive screening above that of the average-risk individual. We did so by identifying and reviewing the medical histories of those enrolled in the Familial Bowel Cancer Service at The Royal Melbourne Hospital and evaluating the rates of polyps and CRC found in this population.

Our primary outcome measures of interest were prevalence rates of CRC and neoplastic polyps. Our secondary outcomes measures of interest were related to the primary outcomes and included age of onset in the progeny of the affected parents (children) – the registrants - and relationship of the CRC and neoplastic polyps to potential risk factors such as (1) gender, (2) age of parental colorectal cancer onset, and (3) presence of neoplasms in siblings. We hypothesized that individuals with two parents that have been affected by colorectal cancer have an increased risk of CRC and neoplastic polyps, at a younger age of onset than the average-risk population.

## Methods

### Study design

We conducted a single-centre retrospective cohort-study of adults (18 years of age and older) prospectively enrolled in the Bowel Cancer Surveillance Service at The Royal Melbourne Hospital. Upon registration in this service, patients gave consent for recording of their individual and familial medical history as pertaining to colorectal cancer in the FamBIS database, the Cancer Council’s central software which supports Victorian Familial Cancer Centres to identify patients with this profile. The study was approved within the ethics framework of the previous studies comparing outcomes in high risk groups [12]. This current study was reviewed by the Human Research Ethics Committees via the Quality Assurance review process and met criteria for a Quality Assurance/Negligible Risk Research project, with ethics approval granted (Project Number QA2016055).

### Study population

The Surveillance database was started in 1980 in order to offer bowel cancer screening services to those felt to be at a higher risk of CRC due to their family history, and upon enrollment, patients were designated into risk categories dependent upon family history. This database was incorporated into FamBIS as the risk management (surveillance) tool for colonoscopy and FIT testing for moderate to high risk individuals. Patients were referred to this service either by their general practitioner or, later when established, through the Familial Cancer Clinic. Individual consent was obtained for each registrant, giving permission to record their individual and familial medical history as pertaining to CRC diagnosis, screening, and surveillance. Risks of procedures were reviewed with patients at each encounter by the performing practitioner as part of informed consent for the procedure. Patients were free to withdraw at any time. Family members’ history and family member’s cancer diagnoses were verified through the comprehensive Cancer Council registry. Such verification was held confidential to the service and not disclosed unless publicly known across the family.

For those classified as having a strong family history of CRC, recommendations were given for screening, including annual FOBT stool testing and colonoscopy every 3–5 years, commencing at age 40 years or 10 years before the earliest age of diagnosis in a close relative, whichever was the earlier. Registration into this database ended in 2010, though screening and surveillance results of these individuals continue to be updated on existing registrants. Those who followed with a local gastroenterologist were free to follow the recommendations of their local practitioner. Colonoscopy could be carried out either at our institution or locally. Follow-up reminders for recommended scheduled screening were sent by mail or through telephone, with results of any testing performed (here or locally) verified and recorded in the FamBIS Surveillance database. Any new findings, symptoms, or events associated with screening were managed under standard practices, including recommendations for follow-up colonoscopy in the event of positive FOBTs.

Using the FamBIS database, we queried the population of enrolled registrants with two parents known to be affected by CRC, with chart review of both paper and electronic records for screening and surveillance results available from 1980 to 2015.

### Exclusion criteria

For our study, we excluded registrants whose parents were not found on verification to have colorectal cancer (e.g. those whose cancers were non-adenocarcinoma-type of colorectal origin, those with large polyps found not to be cancerous), registrants with no screening results available, registrants who were in families identified by genetic testing to have a familial colorectal cancer syndrome or known genetic mutation, and those with inability to give informed consent, understand English, or with contraindication to screening/surveillance procedures. We also excluded patients with other family history, including patients with siblings with colorectal cancer.

### Data collection

Data collected on registrants included family history (including colorectal cancer verification), demographic information, and screening/surveillance dates and results (including fecal occult blood test stool results, colonoscopy and pathology reports, and imaging results such as CT colonography and barium-contrast barium enema, as well as genetic testing results if available (for exclusion purposes). We defined neoplasms as all tubular adenomas [TA], sessile serrated adenomas/polyps [SSA/P], mixed adenomatous pathology, and including advanced neoplasms. Advanced neoplasms were defined as adenomas with size 1 cm in size or greater as reported by endoscopist or pathology with 20% or more villous component or high-grade/severe dysplasia, as well as adenocarcinomas (CRC). Sessile serrated adenomas/polyps were only considered advanced if they had a component of dysplasia. When available, we recorded the most advanced or “worst” pathology of all neoplasms, with the worst being CRC, followed by advanced neoplasms, and finally other non-advanced neoplasms. For those in whom only pathology reports were available for review, polyp size was determined by the pathology specimen report, with the understanding that measurements in pathologic specimens are usually smaller than that reported by the endoscopist due to formalin and other fixing methods used in the preparation of the specimen.

### Statistical analysis

While the FamBIS database included identifiable data linked by database-specific identification numbers (given the need to be able to verify and update results), the analysis performed for this study was conducted using de-identified study-specific IDs.

We calculated *p*-values based on chi-square test for categorical variables and Fisher’s Exact test for small values, as well as t-test for continuous variables. Relative risks [RR] compared to average-risk populations were used to calculate number needed to treat [NNT], with confidence intervals [CI] also reported. We conducted Kaplan-Meier and log-rank survival analysis for our secondary outcomes.

## Results

### Registrant demographics

**Fig. 1:**
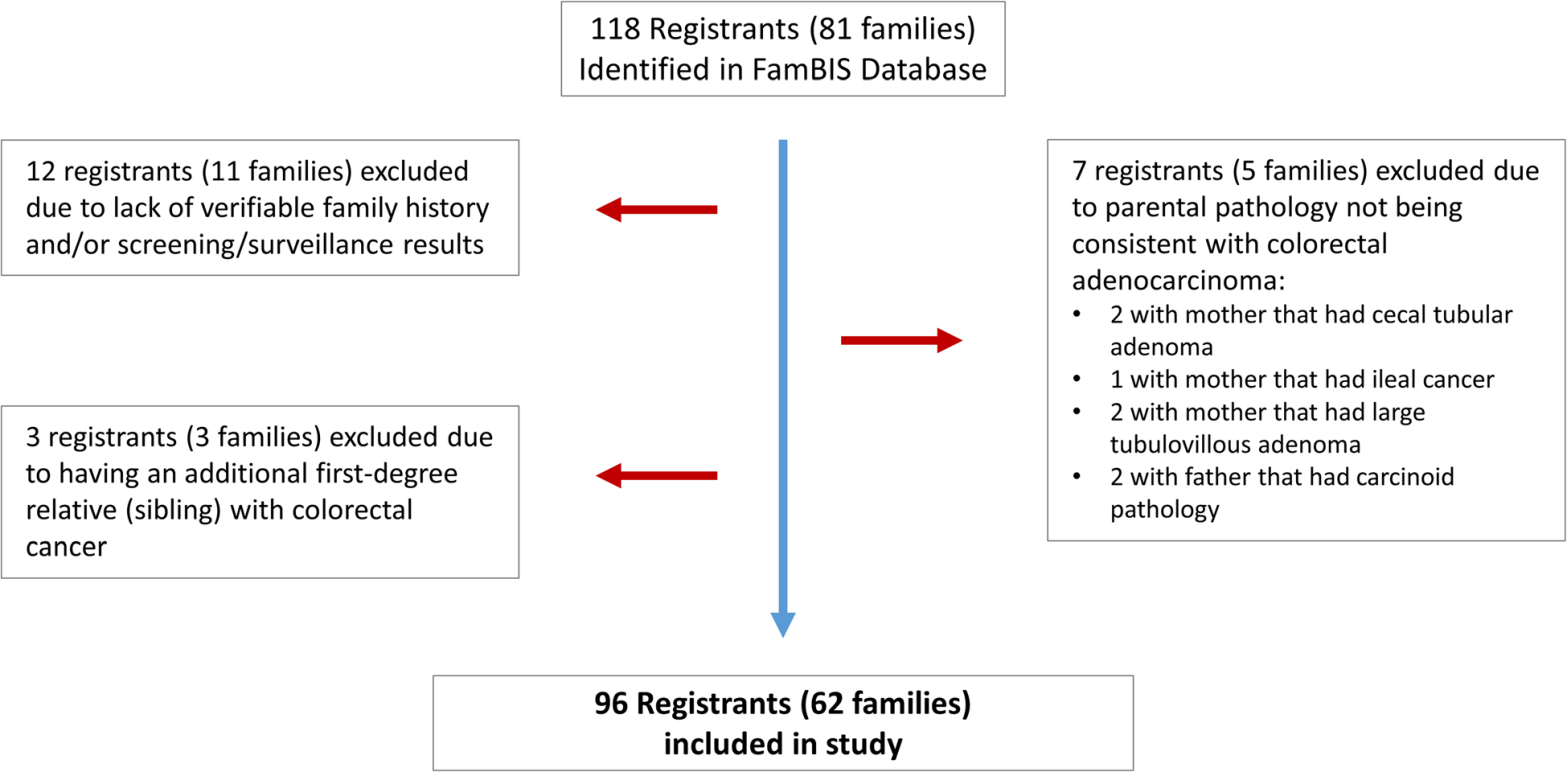
Registrant exclusions

A total of 118 registrants whose both parents had CRC were collected from the database, with 7 registrants excluded after review of pathology reports of parents not being consistent with adenocarcinoma, and an additional 12 registrants excluded for lack of verifiable family history or screening information available (Fig. 1). Three registrants were excluded due to the history of CRC in at least one sibling. Final analysis for our study was conducted on the remaining 96 registrants from 62 unique families.

Overall, 58.3% of registrants were female (Table 1). Registrants began screening as early as 24 years of age and as late as 70 years of age, with the mean age of first screening being at 44.6 ± 10.7 years old. The mean duration of screening/surveillance was 17.3 ± 10.1 years, with the oldest registrant receiving surveillance until 87 years old. Through their screening period, registrants underwent a mean of 11.5 ± 9.1 FOBTs and 4.4 ± 3.1 colonoscopies or sigmoidoscopies. Given the time period of our study, computed tomography [CT] colonography was utilized by only one registrant, with reportedly normal study results.

**Table 1:** Demographics of Registrants.

Characteristic	All Registrants (*N* = 96)
Demographics	
Gender - Female	56 (58.3%)
Age at first screening (years)	44.6 ± 10.7
Age at first FOBT (years)	45 ± 10.4
Age at first colonoscopy (years)	46 ± 10.4
Duration of screening/surveillance (years)	17.3 ± 10.1
Number of FOBTs per registrant	11.5 ± 9.1
Number of colonoscopies/sigmoidoscopies per registrant	4.4 ± 3.1
Family History	
Age of Father at colorectal cancer diagnosis	64.9 ± 8.8
Age of Mother at colorectal cancer diagnosis	64.6 ± 10.5
At least one parent diagnosed at < 55	25 (26.0%)
At least one sibling with advanced neoplasm	7 (7.3%)
At least one sibling with neoplasm	32 (33.3%)

Plus-minus values are means ± SD

The mean age of colorectal cancer diagnosis in the registrants’ parents were similar for both the father (64.9 ± 8.8) and mother (64.6 ± 10.5). Only a minority of registrants (26.0%) had at least one parent diagnosed with young-onset (< 55 years of age) CRC, the balance having both parents diagnosed with CRC 55 years age or older. One-third (33.3%) of registrants had at least one sibling with neoplasms of any type, of whom 7 registrants (7.3%) had at least one sibling with an advanced neoplasm (Table 1).

### Registrant findings

Over the course of screening, 41 (42.7%) registrants were found to have at least one neoplasm of any kind (including adenomas, advanced adenomas, and CRC) as their first positive colonoscopic finding (Table 2). Overall prevalence rates for findings were: all adenomas (39.6%), non-advanced adenomas (30.2%), advanced adenomas (10.4%), and CRC (2.1%).

**Table 2:** Registrant Findings

Characteristic	Registrants
Overall Prevalence Rates	*N* = 96
All Neoplasms	41 (42.7%)
All Adenomas	39 (40.6%)
Non-Advanced Adenomas	29 (30.2%)
Advanced Adenomas	10 (10.4%)
Colorectal cancer [CRC]	2 (2.1%)
Registrant Neoplasms	*N* = 41
Pathology for First Neoplasms	
Tubular Adenoma [TA]	17 (41.4%)
Sessile Serrated Adenoma/Polyp [SSA/P]	11 (26.8%)
Mixed adenomatous pathology	3 (7.3%)
Pathology unspecified	1 (2.4%)
Advanced neoplasm	9 (21.9%)
Tubulovillous adenoma [TVA]	4 (9.8%)
Large polyp--TA	3 (7.3%)
CRC	2 (4.9%)
Mean age of first neoplastic finding	53.5 ± 11.0
Number of young-onset first neoplasms	22 (53.7%)
FOBT positive within 6 months prior (out of all colonoscopies with neoplasms, n = 61)	9 (14.8%)
Registrant Advanced Neoplasms	*N* = 12
Pathology for First Advanced Neoplasms	
TVA	5 (41.7%)
Large polyp--TA	5 (41.7%)
CRC	2 (16.7%)
Mean age of first advanced finding	54.1 ± 9.44
Number of young-onset advanced neoplasms (out of all advanced neoplasms, n = 12)	6 (50.0%)
Registrant Colorectal Cancers	
Mean age of colorectal cancer diagnosis	64.5 ± 5.5

Plus-minus values are means ± SD

Percentages may not add up to 100% due to rounding

The mean age of first neoplastic finding was 53.5 ± 11.0, with over half (53.7%) found at < 55 years of age. Of these registrants’ first neoplastic findings on colonoscopy, 9 (21.9%) were advanced neoplasms, which included tubulovillous adenomas [TVA] (9.8%), large TAs (7.3%), and CRC (4.9%). While for most registrants, the first positive screening colonoscopy was found to harbor the most advanced neoplasm over their surveillance period, 3 patients who initially had only non-advanced neoplasms on their first positive screening colonoscopy were subsequently found to have advanced neoplasms on a surveillance colonoscopy. Interestingly, out of all colonoscopies in which neoplasms were identified (*n* = 61), only 9 (14.8%) had an FOBT that was positive within 6 months prior.

In total, 12 (12.5%) of the registrants were found to have an advanced neoplasm over the course of screening and surveillance (Table 2). The mean age of first advanced neoplasm was similar to the mean age of first neoplastic findings overall at 54.1 ± 9.44, with half (50.0%) found at < 55 years of age. The breakdown of registrants’ first advanced neoplastic findings included TVAs (41.7%), large TAs (41.7%), and CRC (16.7%). In our cohort, there were only 2 patients found to be diagnosed with CRC, and of note, these 2 registrants were diagnosed prior to registration in the database (Table 2), at 59 and 70 years of age.

### Characteristics of registrants with neoplasms found during screening/surveillance

There was a significantly lower proportion of females in registrants with neoplasms compared to those without (45.0% vs 67.8%, *p* = 0.0251), and while this trend was also noted in registrants with advanced neoplasms (45.5% vs 60.0%, *p* = 0.3572), this was not statistically significant (Table 3). Registrants with neoplasms had a trend towards older age at first colonoscopy than those without neoplasms (46.2 ± 10.6 vs 43.4 ± 10.7), but this was not statistically significant (*p* = 0.2009). Those with neoplasms, however, had a longer mean duration of interval between colonoscopies by approximately 5 years (20.3 ± 9.0 vs 15.1 ± 10.4, *p* = 0.0107), which likely accounted for the higher number of colonoscopies and sigmoidoscopies performed (5.8 ± 2.7 vs 3.4 ± 3.0, *p* = 0.0001) and reciprocally, the longer screening duration would allow a greater opportunity for neoplasia to be detected. This trend was also noted in registrants with advanced neoplasms as compared to those without advanced neoplasms (6.5 ± 2.6 vs 4.1 ± 3.0, *p* = 0.0125), though there was no significant difference in duration of screening (*p* = 0.2220). This highlights the difficulty of equating long interval with advanced pathology, and carries a number of well described explanations. There was no significant difference in the number of iFOBTs used for screening in any of the subgroups. The only deaths reported during the period of the surveillance period were in the two registrants who had been diagnosed with CRC and enrolled in the database retroactively.

**Table 3:** Subgroup analysis of registrants with neoplasms or advanced neoplasms

Characteristic	No	All		No Advanced	Advanced	
	Neoplasms	Neoplasms	*p*-	Neoplasms	Neoplasms	
	(*N* = 56)	(*N* = 40)	value*	(*N* = 85)	(*N* = 11)	*p*-value*
Gender - Female	38 (67.8%)	18 (45.0%)	**0.0251**	51 (60.0%)	5 (45.5%)	0.3572
Screening/Surveillance						
Age at first screening (years)	43.4 ± 10.7	46.2 ± 10.6	0.2009	44.2 ± 10.8	47.5 ± 10.0	0.3329
Duration of screening (years)	15.1 ± 10.4	20.3 ± 9.0	**0.0107**	16.9 ± 10.4	20.2 ± 7.8	0.2220
Number of FOBTs	10.6 ± 9.3	12.8 ± 8.8	0.2393	11.2 ± 9.2	13.7 ± 7.1	0.3050
Number of colonoscopies/sigmoidoscopies	3.4 ± 3.0	5.8 ± 2.7	**0.0001**	4.1 ± 3.0	6.5 ± 2.6	**0.0125**
Family History						
Age of Father at colorectal cancer diagnosis	65.8 ± 8.2	63.5 ± 9.6	0.2317	64.7 ± 9.0	66.2 ± 7.4	0.5529
Age of Mother at colorectal cancer diagnosis	64.9 ± 10.7	64.2 ± 10.2	0.7478	64.3 ± 10.8	66.5 ± 7.2	0.4140
At least one parent diagnosed at < 55	17 (30.4%)	9 (20.0%)	0.2543	24 (28.2%)	1 (9.1%)	0.1734
At least one sibling with advanced neoplasm	5 (8.9%)	2 (5.0%)	0.4655	6 (7.1%)	1 (9.1%)	0.8073
At least one sibling with neoplasm	16 (28.6%)	16 (40.0%)	0.2416	27 (31.8%)	5 (45.5%)	0.3648

* *p*-value based on t-test for continuous variables and chi-square for binary variables, with *p*<0.05 considered statistically significant

Plus-minus values are means ± SD

### Family history of registrants

The ages of CRC diagnosis in the registrants’ parents did not appear to be significantly different in subgroups of those with neoplasms or advanced neoplasms compared to those without (Table 3). Registrants with advanced neoplasm, however, were also more likely to have at least one sibling with advanced neoplasm (16.7% vs 7.4%, *p* = 0.3582) or neoplasm (50.0% vs 30.9%, *p* = 0.1696).

### Survival analysis

**Fig. 2:**
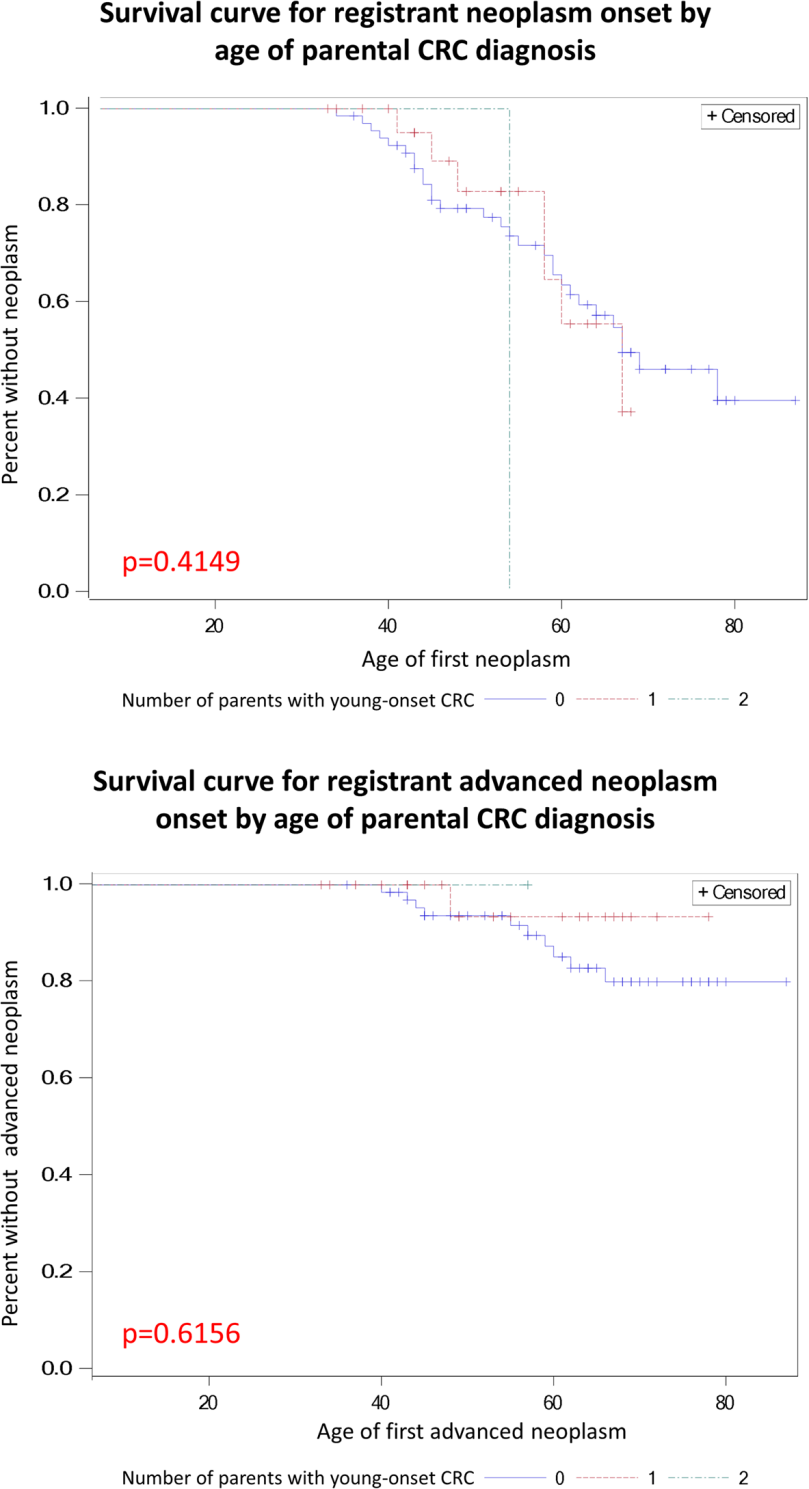
Survival curve for neoplasm onset by parental CRC diagnosis

We further evaluated the relationship of parental and sibling colorectal cancer diagnoses on age of neoplasm onset in the registrants. When stratifying by the presence of young-onset CRC (age < 55) in a registrant’s parent(s), contrary to expectations, we found that registrants with no parents with young-onset CRC initially had a trend towards earlier onset of first neoplasm and advanced neoplasm, compared to those registrants with 1 or 2 parents with young-onset CRC (Fig. 2). These trends, however, reversed for first neoplasm onset by age 60, and differences were not found to be statistically significant for registrant age of onset of first neoplasm (*p* = 0.41) or advanced neoplasm (*p* = 0.62).

## Discussion

While many previous studies have explored the association of family history (and specifically first-degree relatives of the proband) and colorectal polyp and cancer prevalence, no other studies have specifically analyzed the outcomes of asymptomatic individuals who underwent screening due to a family history with both parents affected by colorectal cancer. Our study evaluated the prevalence of colorectal neoplasms in this subpopulation as followed in a single-centre screening program over a 35 year period.

In Taylor et al’s seminal study on colorectal cancer risk and family history, the phenomenon of having two affected parents was discussed, though it was found that the familial relative risk [FRR] of colorectal cancer in those with both an affected mother and an affected father (FRR 4.97; 95% CI 2.72–8.34)—while increased—was not significantly so (*p* = 0.07) over those with any two affected first-degree relatives (FRR 3.21; 95% CI 2.87–3.58) when parents were excluded [13].

There are few studies that have looked at population-based prevalence rates for adenomas worldwide in average-risk populations, and these have found wide ranges in prevalence from region to region [14–19]. Adenoma prevalence rates found by colonoscopy in these studies ranged from 13.7–30.7%, while advanced adenoma rates ranged from 2.15–10.4%, and CRC rates ranged from 0.16 to 1.5%. Calculations of relative risk depend on the prevalence rates of comparative average-risk populations. While there are studies on adenoma detection rates in Australia, there are none on neoplasm prevalence rates in average-risk populations, as colonoscopy is not funded as a screening modality for average-risk Australians. Therefore, we chose to calculate relative risks [RR] of colorectal neoplasms based on the prevalence rates presented by Heitman et al., who performed a meta-analysis to determine pooled prevalence rates based on North American average-risk population studies. In their study, the prevalence rates for neoplastic findings were: adenomas (30.2%), non-advanced adenomas (17.7%), advanced adenomas (5.7%), and CRC (0.3%). Our prevalence rates and calculated relative risks were: adenomas 40.6% (RR 1.3, CI 1.0–1.7, *p* = 0.03, NNT 10), non-advanced adenomas 30.2% (RR 1.7, CI 1.2–2.4, *p* < 0.01, NNT 8), advanced adenomas 10.4% (RR 1.8, CI 1.0–3.5, *p* = 0.06, NNT 21), and CRC (RR 6.9, CI 1.2–41.1, p = 0.03, NNT 56). All neoplastic findings in our population had relative risks that were statistically significant when compared to Heitman et al. While it is difficult to draw conclusions on the rates of CRC in our population given the small sample size and wide confidence intervals, the increased relative risk of adenomas (both advanced and non-advanced), with overall low NNT suggests that our study subpopulation is at a higher risk for pre- and early-cancerous neoplasms, compared to average-risk populations.

Therefore, we believe there is a role for more intensive screening and surveillance in this subpopulation beyond that recommended for the average-risk population, though the optimal screening modality (colonoscopy vs FIT/iFOBT) and intervals for higher risk populations is still the subject of many other studies at this time. In the United States, colonoscopy is considered a first-line screening modality and is the only recommended modality for those at increased risk of CRC, with advantages of being able to not only detect pre- and early-cancerous neoplasms, but also allowing for possible cancer prevention through interventions such as endoscopic resections of such lesions. However, it is an invasive and expensive procedure with some complication risk, as well as limited adherence. On the other hand, in our cohort, we found that of all colonoscopies in which neoplasms were identified (*n* = 61), only 9 (14.8%) registrants had an FOBT that was positive within 6 months prior to the colonoscopy, despite annual FOBTs. This further suggests that many of these registrants with neoplasms would have been missed under the average-risk individual categorization and with use of FOBT alone for screening. We do note that in our study population only guaiac-based FOBT was available for screening during the study period, nowadays screening is recommended with FIT/iFOBT which has a better sensitivity and is considered a superior screening test. In a meta-analysis of the diagnostic performance of FIT/iFOBT for detecting CRC or advanced neoplasia [AN] in patients at above-average personal or familial risk, however, it was found that FIT has high overall diagnostic accuracy for CRC but only moderate accuracy for AN [20]. In addition, while Quintero et al’s study of annual FIT for detection of AN vs colonoscopy in asymptomatic patients with a family history of CRC found annual FIT to be as good as colonoscopy for diagnosing AN, it still missed almost 40% of advanced adenomas [21]. The data from these studies suggest that in a population with increased risk of CRC, while annual FIT/iFOBT could be used as a screening modality for CRC, colonoscopy may have a higher yield in cancer prevention by diagnosing and intervening on pre-cancerous lesions, while FIT/iFOBT may serve better as an alternative screening option in those patients who refuse colonoscopy. Future studies are needed to better optimize guidance on screening in this population.

Regarding our secondary outcomes, we found that neither gender, the age of parental CRC diagnosis, nor the presence of neoplasms in the sibling was statistically significant for the natural history of neoplasm development in the registrant, though there was a trend towards a higher proportion of registrants with neoplasms or advanced neoplasms having at least one sibling with a neoplasm. As suggested by Taylor’s paper, this makes sense genetically as predisposing genes segregate in the offspring of each affected parent, which therefore increases risk in the proband generation over that seen in the individual parents alone. This is also corroborated by findings in a cross-sectional study from Hong Kong which found that siblings of individuals with at least 1 advanced adenoma had a 6-fold increased odds of advanced adenoma compared with subjects whose siblings had no identified neoplasia [22].

One limitation of our study is that the registrants were from a single tertiary care centre, and as such the findings may not be generalizable to other populations in Australia or around the world. In addition, because registrants were free to undergo the recommended screening tests at local facilities, and then forward their results for inclusion in the FamBIS database, there was also risk of missing or incomplete data despite attempts by database managers to request and acquire complete data when possible. Also, while attempts were made to verify family history of colorectal cancer and/or neoplasms through medical records and death certificates, this was not always possible due to registrant family members being in other states, territories, or countries. Our study did not include a control group, let alone any randomization, thus representing a small cohort study. Comparisons of risk have been made against contemporary colonoscopy studies of average-risk subjects necessarily outside Australia due to such average-risk colonoscopy screening in Australia being not supported by guidelines or funding. Another issue was that, due to the small number of patients in our study population, we were under-powered to describe the natural history of CRC alone, as only two patients in the registry were noted to be diagnosed with CRC.

Overall, our findings correspond with the updated 2017 NHMRC-approved guidelines in which patients in our study population would now be considered at least Category 2, with two first-degree relatives with colorectal cancer diagnosed at any age [11]. For these patients, screening strategies now include iFOBT every 2 years from age 40 to 50 and colonoscopy every 5 years from ages 50 to 74. In addition, those with two parents, as well as at least one sibling with CRC, have a higher risk of advanced neoplasms as well, and appropriately are now considered Category 3 risk, with even earlier starting ages recommended for iFOBT and colonoscopy screening. Our study also emphasizes the importance of being able to collect reliable family history as well as accurate risk prediction based on family history, in order to best guide patients towards appropriate and effective prevention and early detection of colorectal cancer.

## Conclusions

The prevalence rates for neoplasms, advanced neoplasms, and CRC in our current study were statistically significantly higher compared with those seen in average-risk populations. This supports the importance of more intensive screening for this subpopulation in preventing colorectal cancers, as well as pre-and early-cancerous neoplasms.

## Data Availability

Not applicable
